# Gross genomic damage measured by DNA image cytometry independently predicts gastric cancer patient survival

**DOI:** 10.1038/sj.bjc.6605266

**Published:** 2009-09-08

**Authors:** J A M Belien, T E Buffart, A J Gill, M A M Broeckaert, P Quirke, G A Meijer, H I Grabsch

**Affiliations:** 1 Department of Pathology, VU University Medical Center, Amsterdam, The Netherlands; 2 Section of Pathology and Tumour Biology, Leeds Institute of Molecular Medicine, University of Leeds, Leeds, UK

**Keywords:** gastric cancer, image cytometry, flow cytometry, DNA ploidy, prognosis

## Abstract

**Background:**

DNA aneuploidy reflects gross genomic changes. It can be measured by flow cytometry (FCM-DNA) or image cytometry (ICM-DNA). In gastric cancer, the prevalence of DNA aneuploidy has been reported to range from 27 to 100%, with conflicting associations with clinicopathological variables. The aim of our study was to compare the DNA ploidy status measured using FCM-DNA and ICM-DNA in gastric cancer and to evaluate its association with clinicopathological variables.

**Methods:**

Cell nuclei were isolated from 221 formalin-fixed, paraffin-embedded gastric cancer samples. DNA ploidy was assessed using FCM-DNA and ICM-DNA.

**Results:**

A total of 178 (80.5%) gastric cancer samples were classified as DNA aneuploid using FCM-DNA, compared with 172 (77.8%) gastric cancer samples when using ICM-DNA. Results obtained from both methods were concordant in 183 (82.8%) cases (*κ*=0.48). Patients with ICM-DNA diploid gastric cancer survived significantly longer than those with ICM-DNA aneuploid gastric cancer (log rank 10.1, *P*=0.001). For FCM-DNA data, this difference did not reach statistical significance. The multivariate Cox model showed that ICM-DNA ploidy status predicted patient survival independently of tumour-node-metastasis status.

**Conclusion:**

ICM-DNA ploidy status is an independent predictor of survival in gastric cancer patients and may therefore be a more clinically relevant read out of gross genomic damage than FCM-DNA.

Despite its declining incidence, gastric cancer remains the second leading cause of cancer death worldwide, with a very poor prognosis (Parkin *et al*, 2001). In the United Kingdom and the Netherlands, it ranks fifth as a cause of cancer death, with 7980 (UK figure from 2005, data obtained from http://info.cancerresearchuk.org/cancerstats/types/stomach/?a=5441) and 2000 (NL figure from 2005, data collected from http://www.ikcnet.nl/page.php?id=1868&nav_id=114) new patients diagnosed with gastric cancer annually. A complete surgical resection of the primary cancer and all potentially involved lymph nodes is the only way to potentially cure the disease. However, <30% of patients are diagnosed with the disease at a resectable stage in the United Kingdom (data obtained from http://www.rcseng.ac.uk/rcseng/content/publications/docs/national-audit-of-oesophago-gastric-cancer-report-2008). At present, the pathological TNM (tumour-node-metastasis) classification is considered as the ‘gold standard’ for predicting patient survival after surgical resection (Sobin and Wittekind, 1997; Klein *et al*, 2001). However, patients with gastric cancers of similar TNM stage show a large variation in survival. Therefore, it seems to be necessary to identify markers that help to better characterise gastric cancer at a molecular level and therefore, increase the precision of prognosis prediction for individual patients.

A number of studies have been published investigating DNA ploidy in gastric cancer, with contradicting results regarding the relationship of DNA ploidy status and tumour stage or patient survival (Brito *et al*, 1993; Suh and Min, 1993; Abad *et al*, 1998; Baba *et al*, 2002; Grabsch *et al*, 2004), which is most likely because of the small number of gastric cancers within studies and the different technologies used.

The DNA ploidy status can be measured either using flow cytometry (FCM-DNA) or image cytometry (ICM-DNA). The DNA ploidy status measured using flow cytometry is relatively fast and has a high measurement precision, allowing the identification of small deviations in DNA content (i.e., DNA index) within a large population of tumour cell nuclei, as usually up to 50 000 cell nuclei are assessed. As only nuclei in suspension are measured using FCM-DNA, one of the main disadvantages is the lack of visual control of the measured object and the related lack of option of ‘artefact rejection’, as the morphological information of the cell nucleus cannot be assessed. In addition, relatively large amounts of tissue are required to prepare the suspension of nuclei, and repeating the measurement using the same suspension is technically challenging. Moreover, low-frequency DNA abnormalities may be missed as they can be obscured in the density profile (Cornelisse and Van Driel-Kulker, 1985). The DNA ploidy status measured using image cytometry overcomes some of these disadvantages by allowing the visual control as well as the selection of nuclei on the basis of morphologically or additionally measured features, such as nuclear shape and texture. In addition, ICM-DNA allows additional or repeat measurements, because the specimen is a fixed and stained nuclear suspension deposited onto a glass slide, which can be kept and stored indefinitely. The main disadvantage of ICM-DNA is the lower throughput, that is, the number of nuclei that can be measured in a given time, which affects the number of cases that can be assessed per day.

The aim of this study was to determine the prognostic value of DNA ploidy status in gastric cancer, measured by both FCM-DNA and ICM-DNA, and to analyse the relationship of DNA ploidy status with clinicopathological variables and patient survival.

## Materials and methods

### Patients

A total of 221 patients with primary gastric cancer, who underwent gastrectomy with curative intent and D2 lymph node dissection at the Academic Department of Surgery at the Leeds General Infirmary (Leeds, UK) between 1970 and 2004, were included in this study. None of the patients received neoadjuvant or adjuvant systemic treatment or radiotherapy. The clinicopathological data are summarised in Table 1. The study was approved by the Local Research Ethics Committee (LREC No. CA01/122).

### Tissue processing

Representative formalin-fixed, paraffin-embedded tissue blocks were retrieved from the pathology archive of the Department of Histopathology (Leeds General Infirmary). ‘Sandwich’ sections of 4-*μ*m thickness were cut and stained with haematoxylin and eosin to confirm the presence of gastric cancer. Nuclear suspensions were prepared from 50-*μ*m-thick sections according to the Hedley procedure (Hedley *et al*, 1983). Part of the nuclear suspension was stained with DAPI (4′,6-diamidino-2-phenylindole, Partec Instruments, Muenster, Germany) for FCM-DNA, and cytospins were prepared from the other part by centrifugation of the specimen for 15 min at 3000 r.p.m. and staining using the Feulgen method according to the consensus of the European Society for Analytical Cellular Pathology (Haroske *et al*, 2001), with minor modifications. In short, cytospin preparations were incubated with 5 N HCl for 30 min at 27 °C, rinsed in distilled water for 5 min, stained with fresh Schiff's reagents for 45 min, and then washed in running tap water for 15 min. The cytospin slides were then dehydrated and coverslipped.

### Measurement of FCM-DNA

FCM-DNA measurements were determined within 3 h after DAPI staining with a Partec PAS II mercury lamp-based flow cytometer (Partec Instruments), using trout erythrocytes as external control cells. The procedure is described in detail elsewhere (Bergers *et al*, 1996).

### Measurement of ICM-DNA

The DNA content of stained nuclei was measured and analysed by ICM-DNA according to a published protocol (Rijken *et al*, 1999). The guidelines of the consensus report of the European Society for Analytical Cellular Pathology (Haroske *et al*, 2001) were followed. Before every image analysis session, Köhler illumination was applied, and the camera was switched on at least 15 min before measurement to ensure standardised conditions. Images were linearly corrected for shading with two empty images, namely one illuminated and one dark-current image (Tenkate *et al*, 1993). The resulting corrected grey values provided a measure for the local optical density. Segmentation was carried out in a fully automatic manner on the basis of the algorithm described by Vossepoel *et al* (1979), and a filter to remove debris and aggregates was active during measurement.

If available, at least 2000 nuclei were measured in a fully automatic manner, and lymphocytes and fibroblasts were included as internal DNA diploid controls. Using classification algorithms, ellipsoid objects resembling fibroblasts, and round dark condense objects resembling lymphocytes, were automatically identified. These two object classes were used as internal controls and to calibrate and scale the DNA histogram. Another set of classification algorithms was applied to automatically remove the majority of remaining debris and aggregates from the data set. The resulting DNA histograms were visually inspected and nuclei, which should have been removed on the basis of features such as shape and texture automatically, but were missed by the classification algorithms, were removed manually. A median of 1024 high-quality nuclei (range: 204–1744) was measured per case.

By convention in DNA cytometry, nuclear DNA content is measured in relative units ‘c’, in which the DNA content of normal, non-tumour nuclei is set at 2c. In this study, the 2c reference value was determined by taking the mean DNA content measured for nuclei that were identified as fibroblasts and lymphocytes. After establishing the 2c reference value, the histogram was scaled up to 10c with a fixed number of 256 bins to obtain standardised histograms that cover a wide range of c values that potentially occur in populations of tumour nuclei.

### Analysis of DNA histogram

All FCM-DNA and ICM-DNA histograms were analysed using the MultiCycle AV computer programme (Phoenix Flow Systems, San Diego, CA, USA), according to a previously described protocol (Bergers *et al*, 1995).

The DNA index was calculated by dividing the modal channel number of DNA aneuploid peaks by the corresponding number of the DNA diploid peak. In case of only one cell cycle, the DNA index was set at 1.00. On the basis of the DNA index, gastric cancers measured using FCM-DNA were classified into three subclasses on the basis of previously published guidelines (Ormerod *et al*, 1998): DNA diploid (only one cell cycle present), DNA tetraploid (1.9⩽DNA index<2.1), and DNA aneuploid (1<DNA index<1.9 or DNA index⩾2.1). Gastric cancers measured using ICM-DNA were classified into three subclasses on the basis of previously published guidelines (Haroske *et al*, 2001): DNA diploid (only one cell cycle present), DNA tetraploid (1.8⩽DNA index<2.2), and DNA aneuploid (1.1⩽DNA index<1.8 or DNA index⩾2.2).

Gastric cancers measured using ICM-DNA were additionally classified on the basis of the so-called 9c exceeding rate (9c ER) (Haroske *et al*, 2001). The 9c ER is defined as the percentage of cells exceeding a DNA content of 9c and has been previously used for the grading of malignancy (Haroske *et al*, 2001). If a case classified as DNA diploid or DNA tetraploid demonstrated a 9c ER >0, this case was reclassified as DNA aneuploid, as the presence of tumour nuclei with DNA content exceeding 9c is believed to be evidence of aneuploidy (Motherby *et al*, 2002). Using FCM-DNA cytometry, by definition, the 9c ER cannot be determined, as this requires a visual inspection of measured objects to exclude clumping artefacts because of clumped nuclei that could give rise to false calls.

Cases were classified according to four different category definitions:

- Two ‘traditional’ categories as introduced by Hedley *et al* (1993): (i) DNA diploid and (ii) DNA non-diploid (category definition A; Table 2A)
- Three categories (i) DNA diploid, (ii) DNA tetraploid, and (iii) DNA aneuploid (category definition B; Table 2B)
- Two categories: (i) DNA non-aneuploid (DNA diploid or DNA tetraploid tumours) and (ii) DNA aneuploid tumours (category definition C; Table 2C)
- Two categories: (i) DNA non-aneuploid (DNA diploid or DNA tetraploid) with 9c exceeding the rate of zero and (ii) DNA aneuploid or DNA non-aneuploid (DNA diploid or DNA tetraploid) with 9c exceeding rate greater than zero (category definition D; Table 2D). It should be noted that category D can only be used with ICM-DNA data for reasons discussed above. To still be able to evaluate any additional value of 9c ER (a key feature of category D), we compared the outcome of category definition D for ICM-DNA with category definition C for FCM-DNA, as the 9c ER is the only difference between category definitions C and D.

### Statistical analysis

Statistical analyses were performed using SPSS (SPSS Version 15 Inc., Chicago, IL, USA). For discrete variables, the *χ*^2^ test was used, or Fisher's exact test if the expected count in a cell was less than five. The correlation between FCM-DNA and ICM-DNA DNA indexes was evaluated by linear regression analysis. Survival time was measured in years from the time of surgery to death. Patients who died of non-cancer-related causes or who were still alive at the end of the study period were censored. Kaplan–Meier curves were plotted and differences between curves were analysed with the Mantel–Cox test. For univariate and multivariate analyses of the predictive values of variables, the Cox proportional hazard model was used, using enter and remove limits of 0.05 and 0.1. A linear predictor score (LPS) was calculated from the Cox model. Results from fitting a Cox model were presented as the regression coefficient, *B*, the *P*-value of B in the Cox model, and the hazard ratio, exp (*B*). The LPS for patients was calculated as PS_i_=*B*_i_ X_1i_+*B*_2_ X_2i_+…+*B*_*p*_ X_*p*i_ for the variables 1 to *p*. Kaplan–Meier curves of the population separated into a high-, intermediate-, and low-risk tertile by the variable LPS illustrated the prognostic values. *P*-values <0.05 were regarded as significant.

## Results

### Clinicopathological characteristics

Tumour and patient characteristics are presented in Table 1. A total of 142 (64%) patients were men and 79 (36%) were women. The median age of all patients was 71 years, ranging from 34 to 96 years. The morphological tumour type of gastric cancers was classified according to the Laurén classification (Lauren, 1965). Median follow-up time was 21 months (range: 1.2–245.8 months). Univariate analysis confirmed the pT status (*P*<0.001), pN status (*P*<0.001), and clinical stage (*P*<0.001) as significant prognostic factors (Table 1).

### DNA ploidy results

DNA ploidy data were available from all 221 gastric cancer patients for both, FCM-DNA and ICM-DNA (Table 2). The gastric cancers were classified and compared using category definitions A (DNA diploid *vs* DNA non-diploid), B (DNA diploid *vs* DNA tetraploid *vs* DNA aneuploid), and C (DNA diploid or DNA tetraploid *vs* DNA aneuploid) for both methods, FCM-DNA and ICM-DNA.

Category definition D (DNA diploid or DNA tetraploid and 9c ER=0 *vs* DNA aneuploid or DNA diploid or DNA tetraploid and 9c ER>0), for reasons intrinsic to technology, can be used only for ICM-DNA data and not for FCM-DNA data, as described in the ‘Materials and Methods’ section.

### FCM-DNA results

On the basis of category definition A (Table 2A), 178 (81%) gastric cancers were classified as DNA non-diploid and 43 (19%) as DNA diploid. The frequency of DNA non-diploid gastric cancers was higher in elderly patients (*P*=0.02, data not shown) and in patients with a higher number of positive lymph nodes (Table 3). Category definition A did not yield any correlation of DNA ploidy data with other clinicopathological variables, including patient survival.

On the basis of category definition B (Table 2B), 43 (19%) patients with DNA diploid or 10 (5%) with DNA tetraploid gastric cancer survived for a longer time compared with 168 (76%) patients with DNA non-diploid gastric cancer; however, this difference was not significant (log rank of 5.5, *P*=0.06). Category definition B did not yield any correlation of DNA ploidy data with other clinicopathological variables, including patient survival.

On the basis of category definition C (Table 2C), 53 (24%) patients with either DNA diploid or DNA tetraploid gastric cancer survived significantly longer than did 168 (76%) patients with DNA aneuploid gastric cancer (log rank: 5.3, *P*=0.02, hazard ratio 1.8 (95% confidence interval: 1.1–2.9)). The results of the univariate survival analysis of DNA ploidy status are shown in Table 4. Category definition C did not yield any correlation of DNA ploidy data with other clinicopathological variables, including patient survival.

Category definition D is not applicable for FCM-DNA data (see the ‘Materials and Methods’ section).

### ICM-DNA results

On the basis of category definition A (Table 2A), 49 (22%) patients with DNA diploid gastric cancers survived significantly longer than did 172 (78%) patients with DNA non-diploid gastric cancers (Table 4; log rank 10.1, *P*=0.001, with hazard ratio of 2.3 (95% confidence interval: 1.4–3.9)). In addition, a significant association was found between DNA ploidy status and age (*P*<0.001, data not shown), but not for any of the other clinicopathological factors (Table 3).

On the basis of category definition B (Table 2B), 49 (22%) patients with DNA diploid gastric cancer had a similar survival compared with 17 (8%) patients with DNA tetraploid gastric cancer, but they had a significantly longer survival compared with 155 (70%) patients with DNA aneuploid gastric cancer (log rank 13.8, *P*=0.001, with hazard ratios of 1.0 (95% confidence interval: 0.3–3.1) for the DNA tetraploid category and 2.4 (95% confidence interval: 1.4–4.2) for the DNA aneuploid category compared with the DNA diploid category).

On the basis of category definition C (Table 2C), 66 (30%) patients with either DNA diploid or DNA tetraploid gastric cancer had a significantly longer survival compared with 155 (70%) patients with DNA aneuploid gastric cancers (Table 4) (log rank 13.8, *P*<0.001, with hazard ratio of 2.4 (95% confidence interval: 1.5–4.0)).

**Figure 1:**
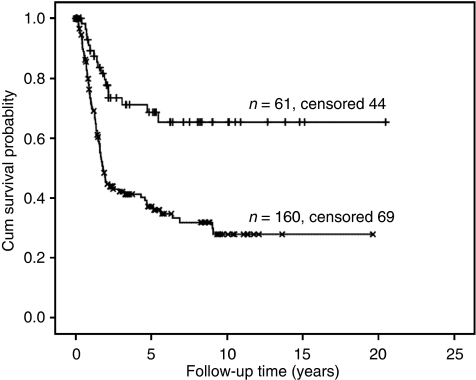
Overall survival related Kaplan–Meier survival curves of patients stratified by DNA ploidy status ICM-DNA diploid+tetraploid (*n*=61) and ICM-DNA aneuploid (*n*=160) gastric cancers taking into account 9c exceeding rate. Log rank: 16.9, *P*<0.001, Hazard ratio: 2.8 (95% confidence interval: 1.7–4.7).

A DNA diploid or DNA tetraploid ICM-DNA histogram, which showed a 9c ER>0, was reclassified as DNA aneuploid, (see the ‘Materials and Methods’ section: category definition D). None of the ICM-DNA diploid but 5 of the 17 (29%) ICM-DNA tetraploid gastric cancers had nuclei with 9c ER>0. These five ICM-DNA tetraploid gastric cancers were therefore reclassified as ICM-DNA aneuploid. On the basis of category definition D (Table 2D), 61 (28%) patients with a DNA diploid or DNA tetraploid gastric cancer had a significantly longer survival compared with 160 (72%) patients with DNA aneuploid gastric cancers (Table 4 and Figure 1) (log rank of 16.8, *P*<0.001, with hazard ratio of 2.8 (95% confidence interval: 1.7–4.7).

### FCM-DNA and ICM-DNA comparison

A significant correlation was observed between the FCM-DNA and ICM-DNA DNA indeces for all DNA ploidy category definitions (*P*<0.001, *r*=0.61).

When comparing DNA diploid with DNA aneuploid gastric cancers (category definition A), results from both methods were concordant in 183 (83%) gastric cancers (*χ*^2^ *P*<0.001, *κ*=0.48; Table 2A and E). In all, 16 (37%) gastric cancers were classified as DNA diploid by FCM-DNA, but as DNA aneuploid by ICM-DNA, and 22 (45%) gastric cancers were classified as DNA diploid by ICM-DNA, but as DNA aneuploid by FCM-DNA.

Using ICM-DNA, the number of DNA tetraploid gastric cancers was higher compared with FCM-DNA (17 and 10 gastric cancers, respectively). When comparing DNA diploid *vs* DNA tetraploid *vs* DNA aneuploid (category definition B), results from both methods were concordant in 168 (76%) gastric cancers (Fisher's exact test: *P*=0.03, *κ*=0.43; Table 2B and E). Interestingly, only three of these gastric cancers were classified as DNA tetraploid by both methods.

When comparing DNA tetraploid gastric cancers combined with DNA diploid gastric cancers with DNA aneuploid gastric cancers (category definition C), results from both methods were concordant in 174 (78.7%) gastric cancers (*χ*^2^ *P*<0.001, *κ*=0.46; Table 2C and E).

As the classification of DNA ploidy into category definition D does not apply to FCM-DNA, we cannot directly compare ICM-DNA category definition D with a similar FCM-DNA category definition. However, to investigate the potential additional value of recognising 9c ER within gastric cancers studied by ICM-DNA, we compared the results from category definition D from ICM-DNA with those from category definition C from FCM-DNA. When comparing ICM-DNA category definition D (ICM-DNA DNA diploid and DNA tetraploid gastric cancers that have a 9c ER>0 (0 and 5 cases, respectively) combined with the DNA aneuploid category *vs* ICM-DNA DNA diploid and DNA tetraploid gastric cancers that do not have a 9c ER>0) with FCM-DNA category definition C (DNA tetraploid gastric cancers combined with DNA diploid gastric cancers *vs* DNA aneuploid gastric cancers), the results were concordant in 177 (80.1%) gastric cancers (*χ*^2^ *P*<0.001, *κ*=0.48; Table 2D and E).

### Multivariate overall survival

Multivariate analyses using statistically significant variables from univariate analyses (Table 1) and the FCM-DNA-based ploidy category definitions (category definitions A, B, and C, Table 2) showed that lymph node status (pN) was the strongest prognostic factor, followed by stage. No other factors including FCM-DNA variables had a significant contribution.

Multivariate analyses using ICM-DNA variables showed that lymph node status (pN), clinical stage, and ICM-DNA ploidy status (using category definition D, Table 2D) were prognostically significant. When dividing the study population in tertiles on the basis of the linear prognostic score, the log rank was 52.8 with *P*<0.001. Hazard ratios for the most favourable group of patients with respect to survival as reference were 3.2 (95% confidence interval: 1.6–6.2) and 8.1 (95% confidence interval: 4.2–15.9), respectively.

## Discussion

Aneuploidy or gross genomic instability is a well-established biological feature of many solid tumours (Lengauer *et al*, 1998; Grabsch *et al*, 2004). At the nuclear level, this gross genomic damage is reflected by abnormal DNA content, for example, DNA aneuploidy, and can be measured using FCM-DNA and ICM-DNA (Friedlander *et al*, 1984; Cornelisse and Van Driel-Kulker, 1985; Mellin, 1990; Ormerod *et al*, 1998; Haroske *et al*, 2001). A wide range of prevalence of DNA aneuploidy has been reported in gastric cancer in the past, and no consensus has been reached regarding the relationship between DNA ploidy status and clinicopathological variables, including patient survival (Grabsch *et al*, 2004). The majority of investigations on the prognostic value of DNA ploidy in gastric cancers have been performed by FCM-DNA (Grabsch *et al*, 2004; Yasa *et al*, 2005; Nesi *et al*, 2007; Doak, 2008) and, to our knowledge, only a single study has used both methods, FCM-DNA and ICM-DNA, in parallel in gastric cancer (Brito *et al*, 1994).

This study aimed at determining whether DNA ploidy status can predict patient prognosis in a large retrospective series of gastric cancer and whether there is a significant difference in DNA ploidy status when measured using available cytometry methods.

FCM-DNA and ICM-DNA are both objective methods to study abnormalities of nuclear DNA content. However, because researchers used different methods to prepare nuclear suspensions and to interpret histograms, published results are difficult to compare, especially with regard to older studies (Brito *et al*, 1994; Grabsch *et al*, 2004; Yasa *et al*, 2005; Nesi *et al*, 2007; Doak, 2008). Lack of standardisation may, at least partially, explain why the published prevalence of DNA aneuploidy in gastric cancer varies so widely (27–100%). To overcome these difficulties, we followed the guidelines of the European consensus report on standardisation of diagnostic DNA image cytometry (Haroske *et al*, 2001) and DNA flow cytometry (Ormerod *et al*, 1998).

Our study demonstrated that FCM-DNA and ICM-DNA were equally sensitive in detecting DNA non-diploid gastric cancers, with the highest overall concordance of 83% of gastric cancers when using the classification system introduced by Hedley *et al* (1993) (‘category definition A’ in this study). The two alternative DNA ploidy classification systems (category definitions B and C) investigated in this study also showed concordance, but at a lower level. The use of the 9c ER to classify gastric cancers as ICM-DNA aneuploid (category definition D) did not increase the percentage of concordant cases between the two methods above the concordance achieved using Hedley's classification.

In the past, only one study (Brito *et al*, 1994) has used both methods in a small group of gastric cancers (*n*=48). Unfortunately, this study does not provide any information regarding the concordance of results using ICM and FCM. The results of this study are comparable with those of previous studies comparing FCM-DNA and ICM-DNA in patients with breast cancer (Ellison *et al*, 1995), in which 75% concordant cases were reported. However, a higher concordance of 91% has been reported in a small number of other cancers (Chen *et al*, 1995).

We can exclude intra-tumour heterogeneity as a possible factor to explain the differences in DNA ploidy classification between FCM and ICM, as FCM-DNA and ICM-DNA analyses were performed using an aliquot from the same nuclear suspension.

**Figure 2:**
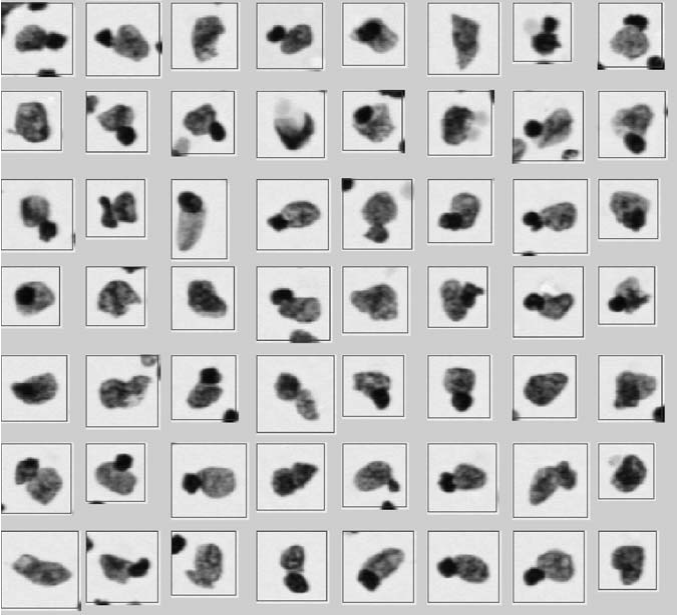
Example objects that could cause a false DNA aneuploid peak detected by FCM-DNA because of clumping of nuclei, whereas ICM-DNA allows the exclusion of cell clumps and artefacts during the visual inspection step of nuclei galleries.

Problems related to different preparation procedures and differences in the interpretation of DNA histograms could potentially explain the discrepancies between the FCM-DNA and ICM-DNA classification found in our study. For example, DNA non-diploid peaks detectable by ICM-DNA may not be visible in FCM-DNA if large numbers of non-tumour DNA diploid nuclei such as those derived from stromal and inflammatory cells are present in the sample at the same time. False DNA tetraploid or aneuploid peaks may be detected by FCM-DNA because of clumping of nuclei, whereas ICM-DNA allows excluding nuclei clumps during the visual inspection step of nuclei galleries (for examples, see Figure 2). DNA non-diploid peaks detected by FCM-DNA, but not by ICM-DNA, could be related to the fact that non-diploid nuclei are more fragile (i.e., they are larger and heavier) and may be more commonly destroyed than DNA diploid nuclei during the centrifugation process in the preparation of cytospin. Although the resolution of ICM-DNA histograms is still slightly lower than that of DNA histograms obtained by FCM-DNA, because of the lower number of nuclei that are measured in ICM-DNA analyses, the resolution in our study has been improved by measuring at least 2000 nuclei compared with measuring typically between 100 and 400 nuclei in the past (Brito *et al*, 1994).

Besides the in-depth study comparing FCM-DNA and ICM-DNA, this study demonstrated that ICM-DNA ploidy status, but not FCM-DNA ploidy status, differs significantly between gastric cancers with different morphology and with different TNM stages, and can stratify patients into different prognostic groups. This is in concordance with some, but not all, previously published studies (Brito *et al*, 1994; Grabsch *et al*, 2004).

In summary, this study showed that patients with DNA non-diploid gastric cancers measured by ICM-DNA have an unfavourable prognosis compared with patients with DNA diploid tumours. Our study demonstrated that ICM-DNA-based DNA ploidy status outperformed FCM-DNA-based DNA ploidy studies in predicting survival in gastric cancer patients. Finally, our results suggest that ICM-DNA DNA ploidy status, taking into account the 9c ER, has an additional value to lymph node status and to clinical stage in predicting the prognosis of gastric cancer patients. Further studies are warranted to investigate whether ICM-DNA ploidy status can be reliably measured in pre-treatment endoscopic biopsies and can potentially been used to stratify patients for treatment.

## Figures and Tables

**Table 1:** Clinicopathological and univariate survival data of 221 patients with gastric cancer

	**All cases *n*=221 (%)**	**Survival *P*-value**		**All cases *n*=221 (%)**	**Survival *P*-value**
*Gender*			*pT status*		
Male	142 (64)	0.49	pT1	14 (6)	**<0.001**
Female	79 (36)		pT2	86 (39)	
			pT3	110 (50)	
*Age*			pT4	11 (5)	
Median (range)	71 (34–96)				
			*pN status*		
*Age categories (years)*			pN0	67 (30)	**<0.001**
<50	12 (5)	0.15	pN1	96 (43)	
50–70	80 (36)		pN2	44 (20)	
⩾70	129 (58)		pN3	14 (6)	
					
*Tumour type*			*pM status*		
Intestinal	147 (67)	0.54	pM0	201 (91)	0.07
Diffuse	44 (20)		pM1	8 (4)	
Mixed	30 (14)		Missing	12 (5)	
					
*Tumour location*			*Clinical stage*		
Cardia	43 (20)	**0.04** a	I	45 (20)	**<0.001**
Body	53 (24)		II	52 (24)	
Antrum	93 (42)		III	85 (39)	
Whole stomach	5 (2)		IV	27 (12)	
Missing	27 (12)		Missing	12 (5)	

Significant correlations are in bold.

a The whole stomach shows shorter survival than does the antrum, body, and cardia separately.

**Table 2:** Comparison of FCM-DNA and ICM-DNA for different DNA ploidy category definitions: (A) traditional DNA diploid *vs* DNA non-diploid category; (B) DNA diploid *vs* DNA tetraploid *vs* DNA aneuploid; (C) DNA non-aneuploid (DNA diploid or DNA tetraploid) *vs* DNA aneuploid; (D) ICM-DNA category i: (DNA diploid or DNA tetraploid) and 9c exceeding rate of 0 *vs* category ii: DNA aneuploid or ((DNA diploid or DNA tetraploid) and 9c exceeding rate >0) *vs* FCM-DNA DNA non-aneuploid (DNA diploid or DNA tetraploid) *vs* DNA aneuploid; (E) Percentage of concordant results, *P*-value obtained using either the *χ*^2^ or Fisher's exact test and the *κ*-values for Tables 2A–D

	**FCM-DNA**			
**(A)**	**Diploid**	**Non-diploid**	**Total**	
*ICM-DNA*				
Diploid	27 (12.2%)	22 (10.0%)	49	
Non-diploid	16 (7.2%)	156 (70.6%)	172	
Total	43	178	221	
				
	**FCM-DNA**			
**(B)**	**Diploid**	**Tetraploid**	**Aneuploid**	**Total**
*ICM-DNA*				
Diploid	27 (12.2%)	1 (0.5%)	21 (9.5%)	49
Tetraploid	5 (2.3%)	3 (1.4%)	9 (4.1%)	17
Aneuploid	11 (5.0%)	6 (2.7%)	138 (62.4%)	155
Total	43	10	168	221
				
	**FCM-DNA**			
**(C)**	**Diploid+tetraploid**	**Aneuploid**	**Total**	
*ICM-DNA*				
Diploid+tetraploid	36 (16.3%)	30 (13.6%)	66	
Aneuploid	17 (7.7%)	138 (62.4%)	155	
Total	53	168	221	
				
	**FCM-DNA**			
**(D)**	**Diploid+tetraploid**	**Aneuploid**	**Total**	
*ICM-DNA*				
Category 1	35 (15.8%)	26 (11.8%)	61	
Category 2	18 (8.1%)	142 (64.3%)	160	
Total	53	168	221	
				
**(E)**	**Concordance (% cases)**	**Pearson's *χ*^2^ *P*-value**	***κ-*value**	
*Category definition*				
A	82.8	*P*<0.001	0.48	
B	76	*P*=0.03^*^	0.43	
C	78.7	*P*<0.001	0.46	
D	80.1	*P*<0.001	0.48	

Abbreviations: FCM-DNA, DNA ploidy status measured using flow cytometry; ICM-DNA, DNA ploidy status measured using image cytometry.

^*^P-value obtained using Fisher's exact test.

**Table 3:** Clinicopathological data and associations with DNA ploidy data

	**All cases**	**FCM-DNA diploid**	**FCM-DNA non-diploid**	**Pearson's *χ*^2^**	**ICM-DNA diploid**	**ICM-DNA non-diploid**	**Pearson's *χ*^2^**
	***n*=221 (%)**	***n*=43 (%)**	***n*=178 (%)**	***P*-value**	***n*=49 (%)**	***n*=172 (%)**	***P*-value**
*Tumour type*							
Intestinal	147 (67)	28 (19)	119 (81)	0.45	32 (22)	115 (78)	0.08
Diffuse	44 (20)	11 (25)	33 (75)		14 (32)	30 (68)	
Mixed	30 (14)	4 (13)	26 (87)		3 (10)	27 (90)	
							
*pT status*							
pT1	14 (6.3%)	4 (29)	10 (71)	0.72	3 (21)	11 (79)	0.99
pT2	86 (38.9%)	16 (19)	70 (81)		19 (22)	67 (78)	
pT3	110 (49.8%)	20 (18)	90 (82)		25 (23)	85 (77)	
pT4	11 (5.0%)	3 (27)	8 (73)		2 (18)	9 (82)	
							
*pN status*							
pN0	67 (30.3%)	18 (27)	49 (73)	**0.009**	17 (25)	50 (75)	0.28
pN1	96 (43.4%)	22 (23)	74 (77)		24 (25)	72 (75)	
pN2	44 (19.9%)	1 (2)	43 (98)		5 (11)	39 (89)	
pN3	14 (6.3%)	2 (14)	12 (86)		3 (21)	11 (79)	
							
*Clinical stage*							
I	9 (4.1%)	8 (18)	37 (82)	0.11	7 (16)	38 (84)	0.23
II	52 (23.5%)	14 (27)	38 (73)		15 (29)	37 (71)	
III	53 (24.0%)	10 (12)	75 (88)		13 (15)	72 (85)	
IV	27 (12.2%)	3 (11)	24 (89)		5 (19)	22 (82)	
Missing	12 (5.4%)	8	4		9	3	

Abbreviations: FCM-DNA, DNA ploidy status measured using flow cytometry; ICM-DNA, DNA ploidy status measured using image cytometry.

Significant associations in bold.

**Table 4:** Univariate survival analyses of FCM-DNA and ICM-DNA for different DNA ploidy category definitions

	**FCM-DNA**			**ICM-DNA**		
**Category definition**	**Log rank value**	***P*-value**	**Hazard ratio (95% confidence interval)^a^**	**Log-rank value**	***P*-value**	**Hazard ratio (95% confidence interval)^a^**
A	2.9	0.09		10.1	0.001	2.3 (1.4–3.9)
B	5.5	0.06		13.8	0.001	1.0 (0.3–3.1)
						2.4 (1.4–4.2)
C	5.3	0.02	1.8 (1.1–2.9)	13.8	<0.001	2.4 (1.5–4.0)
D	NA			16.8	<0.001	2.8 (1.7–4.7)

Abbreviations: FCM-DNA, DNA ploidy status measured using flow cytometry; ICM-DNA, DNA ploidy status measured using image cytometry; NA, not applicable.

Same category definitions as in Table 2. The column hazard ratios of category definition B shows two values. The first value is the hazard ratio of the DNA tetraploid category compared with the DNA diploid nearest ratio category, and the second value is the hazard ratio of the DNA aneuploid category compared with the DNA diploid category.

a Hazard ratio is only presented when significant (confidence interval does not contain 1), and represents values to reference group (i.e., favourable outcome).
